# *N*-[(*E*)-Thio­phen-2-yl­methyl­idene]-1,3-benzothia­zol-2-amine

**DOI:** 10.1107/S1600536812030498

**Published:** 2012-07-18

**Authors:** Irvin N. Booysen, Muhammed B. Ismail, Matthew P. Akerman

**Affiliations:** a School of Chemistry and Physics, University of KwaZulu-Natal, Private Bag X01, Scottsville 3209, Pietermaritzburg, South Africa

## Abstract

In the title thio­phene-derived Schiff base compound, C_12_H_8_N_2_S_2_, the thio­phene ring is slighty rotated from the benzothia­zole group mean plane, giving a dihedral angle of 12.87 (6)°. The largest displacement of an atom in the mol­ecule from the nine-atom mean plane defined by the non-H atoms of the benzothia­zole ring system is 0.572 (1) Å, exhibited by the C atom at the 3-position of the thio­phene ring. In the crystal, weak C—H⋯S hydrogen bonds involving the thio­phene group S atom and the 4-position thio­phene C—H group of a symmetry-related mol­ecule lead to an infinite one-dimensional chain colinear with the *c* axis. The structure is further stabilized by π–π inter­actions; the distance between the thia­zole ring centroid and the centroid of an adjacent benzene ring is 3.686 (1) Å. The crystal studied was an inversion twin with the ratio of components 0.73 (3):0.27 (3).

## Related literature

For the synthesis and crystal structure of 2-amino­benzothia­zole, see: Ding *et al.* (2009 ▶). For crystal structures containing 2-amino­benzothia­zole derivatives, see: Garcia-Hernandez *et al.* (2006 ▶). For inhibitory properties against human cancer cell lines and general anti­tumor properties of benzothia­zole derivatives, see: Racane *et al.* (2001 ▶); O’Brien *et al.* (2003 ▶). For anti­bacterial, anti­fungal, anti­tumor and anti­viral activites of benzthia­zoles, see: Yadav & Malipatil (2011 ▶); Singh & Seghal (1988 ▶); Pattan *et al.* (2005 ▶).
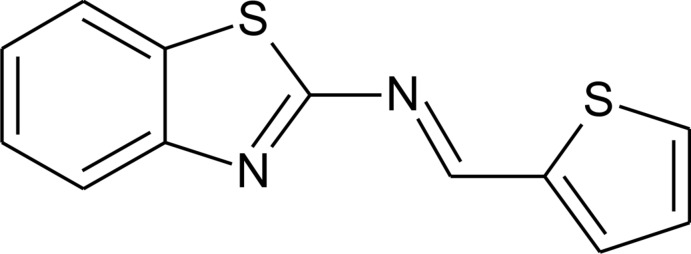


## Experimental

### 

#### Crystal data

C_12_H_8_N_2_S_2_
*M* *_r_* = 244.32
Monoclinic, 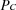

*a* = 10.7244 (5) Å
*b* = 4.6021 (2) Å
*c* = 11.1280 (5) Å
β = 100.367 (2)°
*V* = 540.25 (4) Å^3^
*Z* = 2
Mo *K*α radiation
μ = 0.46 mm^−1^
*T* = 100 K
0.45 × 0.20 × 0.12 mm

#### Data collection

Bruker APEXII CCD diffractometer
Absorption correction: multi-scan (*SADABS*; Bruker, 2010 ▶) *T*_min_ = 0.625, *T*_max_ = 0.749
14476 measured reflections
7768 independent reflections
7126 reflections with *I* > 2σ(*I*)
*R*_int_ = 0.033

#### Refinement

*R*[*F* ^2^ > 2σ(*F* ^2^)] = 0.033
*wR*(*F* ^2^) = 0.085
*S* = 1.04
7768 reflections
145 parameters
2 restraints
H-atom parameters constrained
Δρ_max_ = 0.62 e Å^−3^
Δρ_min_ = −0.36 e Å^−3^
Absolute structure: Flack (1983 ▶), 2974 Friedel pairs
Flack parameter: 0.27 (3)

### 

Data collection: *APEX2* (Bruker, 2010 ▶); cell refinement: *SAINT* (Bruker, 2010 ▶); data reduction: *SAINT*; program(s) used to solve structure: *SHELXS97* (Sheldrick, 2008 ▶); program(s) used to refine structure: *SHELXL97* (Sheldrick, 2008 ▶); molecular graphics: *WinGX* (Farrugia, 1999 ▶); software used to prepare material for publication: *publCIF* (Westrip, 2010 ▶).

## Supplementary Material

Crystal structure: contains datablock(s) I, global. DOI: 10.1107/S1600536812030498/lh5495sup1.cif

Structure factors: contains datablock(s) I. DOI: 10.1107/S1600536812030498/lh5495Isup2.hkl

Supplementary material file. DOI: 10.1107/S1600536812030498/lh5495Isup3.cml

Additional supplementary materials:  crystallographic information; 3D view; checkCIF report

## Figures and Tables

**Table 1:** Hydrogen-bond geometry (Å, °)

*D*—H⋯*A*	*D*—H	H⋯*A*	*D*⋯*A*	*D*—H⋯*A*
C11—H11⋯S2^i^	0.95	2.92	3.517 (1)	122 (1)

Symmetry code: (i) 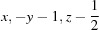.

**Fig. 1.**
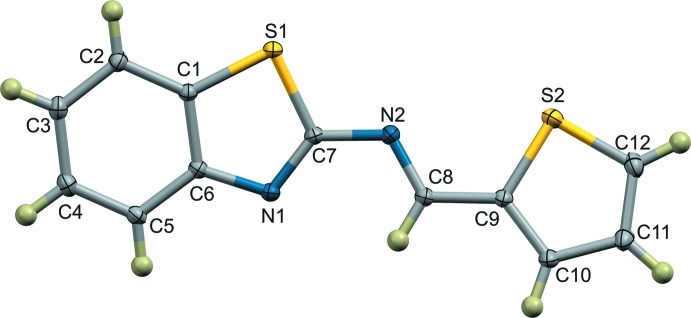
A thermal ellipsoid plot of (I). Ellipsoids are rendered at the 50% probability level.

**Fig. 2.**
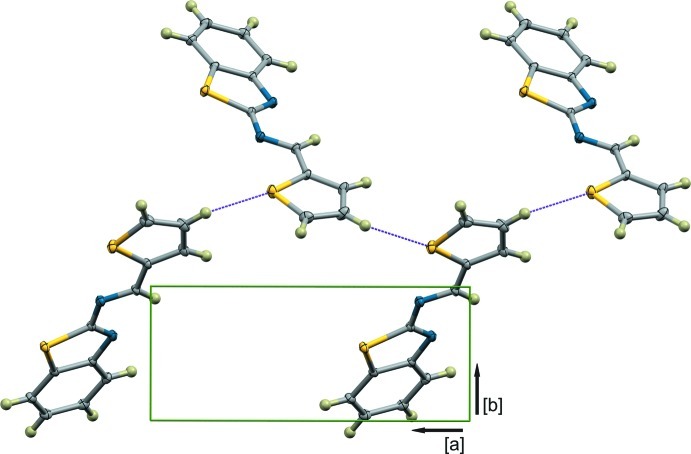
The one-dimensional, hydrogen-bonded chains of (I). Viewed along the a-axis. Hydrogen bonds are shown as dotted lines.

## supplementary crystallographic information

### Comment

Benzothiazoles are naturally occurring molecules which consist of a 5-membered 1,3-thiazole ring fused to a benzene ring. Their derivatives are abundantly distributed in nature and have been shown to have very interesting pharmacological activity, particularly antibacterial, antifungal, antitumor and antiviral properties (Yadav & Malipatil, 2011; Singh & Seghal, 1988; Pattan *et al.*, 2005). The heterocyclic scaffold is readily substituted at the 2-position of the thiazole ring, allowing for derivatization.

The thiophene ring of the title compound (I) is not in the same plane as the 1,3-benzothiazole moiety, with a dihedral angle of 13.6 (1)° relative to the benzthiazole ring. This out-of-plane rotation of the thiophene results in the carbon atom in the 3-position of the thiophene ring (C10) sitting 0.572 (1) Å from the 9-atom mean plane defined by all non-hydrogen atoms of the benzthiazole ring system. The C8—N2 bond distance of 1.290 (1) Å and the C7—N2—C8 bond angle of 118.12 (7)° emphasize the *sp*^2^ hybridization of the imino nitrogen atom (refer to Figure 1 for the atom numbering scheme). An (*E)*-configuration about the imine bond is observed for this Schiff base moiety.

The structure exhibits both hydrogen bonding and π···π interactions. The distance between the centroid of the benzene ring and the centroid of the thiazole ring of an adjacent molecule is 3.686 (1) Å. In addition to the π···π interactions there are non-classical hydrogen bonds between the thiophene sulfur atom, S2, and the thiophene hydrogen atom H11 of an adjacent molecule. This hydrogen bond links the molecules into infinite, one-dimensional hydrogen-bonded chains, which are co-linear with the *c*-axis. The adjacent, hydrogen-bonded molecules are not both in the same plane, the 24-atom mean planes of two adjacent molecules make an angle of 75.9 (1)° to each other. Although the hydrogen bonds are not likely very strong as they are only marginally shorter than the sum of the van der Waals radii (0.066 Å), these intermolecular interactions can stabilize the lattice.

### Experimental

A mixture of 2-aminobenzothiazole (1.27 g; 8.45 mmol) and thiophene-2-carbaldehyde (0.92 ml; 10.2 mmol) in methanol (50 ml) was heated to reflux for 24 h. The resulting orange solution was allowed to cool to room temperature and concentrated by rotary evaporation under reduced pressure. Dry toluene (45 ml) was added to the solution and heated to reflux with a Dean and Stark apparatus for an additional 24 h. Upon cooling the title compound was isolated as brown, needle-shaped crystals.

### Refinement

The positions of all C-bonded hydrogen atoms were calculated using the standard riding model of *SHELXL97* (Sheldrick, 2008) with C—H(aromatic) distances of 0.93 Å and *U*_iso_ = 1.2*U*_eq_(C).

### Crystal data

**Table:**

C_12_H_8_N_2_S_2_	*F*(000) = 252
*M**_r_* = 244.32	*D*_x_ = 1.502 Mg m^−^^3^
Monoclinic, *P**c*	Mo *K*α radiation, λ = 0.71073 Å
Hall symbol: P -2yc	Cell parameters from 7126 reflections
*a* = 10.7244 (5) Å	θ = 1.9–46.7°
*b* = 4.6021 (2) Å	µ = 0.46 mm^−^^1^
*c* = 11.1280 (5) Å	*T* = 100 K
β = 100.367 (2)°	Neelde, yellow
*V* = 540.25 (4) Å^3^	0.45 × 0.20 × 0.12 mm
*Z* = 2	

### Data collection

**Table:**

Bruker APEXII CCD diffractometer	7768 independent reflections
Radiation source: fine-focus sealed tube	7126 reflections with *I* > 2σ(*I*)
Graphite monochromator	*R*_int_ = 0.033
φ and ω scans	θ_max_ = 46.7°, θ_min_ = 2.0°
Absorption correction: multi-scan (*SADABS*; Bruker, 2010)	*h* = −21→21
*T*_min_ = 0.625, *T*_max_ = 0.749	*k* = −9→9
14476 measured reflections	*l* = −21→22

### Refinement

**Table:**

Refinement on *F*^2^	Secondary atom site location: difference Fourier map
Least-squares matrix: full	Hydrogen site location: inferred from neighbouring sites
*R*[*F*^2^ > 2σ(*F*^2^)] = 0.033	H-atom parameters constrained
*wR*(*F*^2^) = 0.085	*w* = 1/[σ^2^(*F*_o_^2^) + (0.0462*P*)^2^ + 0.0113*P*] where *P* = (*F*_o_^2^ + 2*F*_c_^2^)/3
*S* = 1.04	(Δ/σ)_max_ = 0.001
7768 reflections	Δρ_max_ = 0.62 e Å^−^^3^
145 parameters	Δρ_min_ = −0.36 e Å^−^^3^
2 restraints	Absolute structure: Flack (1983), 2974 Friedel pairs
Primary atom site location: structure-invariant direct methods	Flack parameter: 0.27 (3)

### Special details

**Table:**

Geometry. All s.u.'s (except the s.u. in the dihedral angle between two l.s. planes) are estimated using the full covariance matrix. The cell s.u.'s are taken into account individually in the estimation of s.u.'s in distances, angles and torsion angles; correlations between s.u.'s in cell parameters are only used when they are defined by crystal symmetry. An approximate (isotropic) treatment of cell s.u.'s is used for estimating s.u.'s involving l.s. planes.
Refinement. Refinement of *F*^2^ against ALL reflections. The weighted *R*-factor *wR* and goodness of fit *S* are based on *F*^2^, conventional *R*-factors *R* are based on *F*, with *F* set to zero for negative *F*^2^. The threshold expression of *F*^2^ > 2σ(*F*^2^) is used only for calculating *R*-factors(gt) *etc*. and is not relevant to the choice of reflections for refinement. *R*-factors based on *F*^2^ are statistically about twice as large as those based on *F*, and *R*- factors based on ALL data will be even larger.

### Fractional atomic coordinates and isotropic or equivalent isotropic displacement parameters (Å^2^)

**Table:**

	*x*	*y*	*z*	*U*_iso_*/*U*_eq_	
S1	0.784499 (18)	0.45575 (5)	0.332022 (16)	0.01350 (4)	
S2	0.443816 (19)	−0.29395 (6)	0.120085 (18)	0.01726 (4)	
N1	0.84951 (6)	0.36879 (17)	0.11966 (6)	0.01280 (9)	
N2	0.66346 (7)	0.11200 (17)	0.15758 (6)	0.01375 (10)	
C4	1.11614 (8)	0.8815 (2)	0.21064 (8)	0.01636 (12)	
H4	1.1852	0.9670	0.1808	0.020*	
C5	1.03822 (8)	0.68618 (19)	0.13798 (7)	0.01428 (11)	
H5	1.0537	0.6364	0.0591	0.017*	
C6	0.93578 (7)	0.56279 (17)	0.18281 (7)	0.01178 (10)	
C7	0.76580 (7)	0.29951 (17)	0.18671 (7)	0.01226 (10)	
C8	0.63195 (8)	0.02760 (18)	0.04564 (7)	0.01376 (11)	
H8	0.6783	0.0935	−0.0142	0.017*	
C9	0.52640 (7)	−0.16652 (18)	0.01185 (7)	0.01283 (10)	
C10	0.47648 (8)	−0.2709 (2)	−0.10331 (8)	0.01632 (12)	
H10	0.5091	−0.2242	−0.1749	0.020*	
C11	0.37091 (9)	−0.4557 (2)	−0.10202 (9)	0.01923 (14)	
H11	0.3248	−0.5476	−0.1727	0.023*	
C3	1.09458 (8)	0.9554 (2)	0.32807 (9)	0.01656 (12)	
H3	1.1494	1.0901	0.3761	0.020*	
C2	0.99466 (8)	0.83477 (19)	0.37474 (7)	0.01491 (11)	
H2	0.9803	0.8841	0.4540	0.018*	
C1	0.91594 (7)	0.63843 (17)	0.30119 (7)	0.01210 (10)	
C12	0.34282 (9)	−0.4870 (2)	0.01255 (10)	0.01891 (14)	
H12	0.2750	−0.6026	0.0303	0.023*	

### Atomic displacement parameters (Å^2^)

**Table:**

	*U*^11^	*U*^22^	*U*^33^	*U*^12^	*U*^13^	*U*^23^
S1	0.01409 (7)	0.01617 (8)	0.01111 (6)	−0.00243 (6)	0.00462 (5)	−0.00251 (6)
S2	0.01686 (8)	0.02164 (9)	0.01432 (7)	−0.00281 (7)	0.00559 (6)	0.00148 (6)
N1	0.0128 (2)	0.0148 (2)	0.0111 (2)	−0.00094 (18)	0.00313 (16)	−0.00076 (18)
N2	0.0137 (2)	0.0151 (3)	0.0127 (2)	−0.00233 (19)	0.00298 (17)	−0.00149 (18)
C4	0.0137 (3)	0.0163 (3)	0.0195 (3)	−0.0015 (2)	0.0040 (2)	0.0025 (2)
C5	0.0129 (2)	0.0164 (3)	0.0143 (2)	−0.0002 (2)	0.0044 (2)	0.0016 (2)
C6	0.0117 (2)	0.0121 (3)	0.0117 (2)	0.00034 (18)	0.00274 (18)	0.00046 (18)
C7	0.0132 (2)	0.0131 (3)	0.0107 (2)	−0.0009 (2)	0.00263 (18)	−0.00112 (19)
C8	0.0142 (3)	0.0150 (3)	0.0125 (2)	−0.0025 (2)	0.0037 (2)	−0.0016 (2)
C9	0.0126 (2)	0.0142 (3)	0.0122 (2)	−0.0008 (2)	0.00370 (19)	−0.00131 (19)
C10	0.0152 (3)	0.0208 (3)	0.0138 (3)	−0.0033 (2)	0.0047 (2)	−0.0044 (2)
C11	0.0140 (3)	0.0229 (4)	0.0211 (3)	−0.0032 (3)	0.0041 (2)	−0.0073 (3)
C3	0.0140 (3)	0.0156 (3)	0.0195 (3)	−0.0028 (2)	0.0012 (2)	−0.0005 (2)
C2	0.0147 (3)	0.0147 (3)	0.0151 (3)	−0.0013 (2)	0.0020 (2)	−0.0022 (2)
C1	0.0119 (2)	0.0123 (3)	0.0122 (2)	−0.00014 (19)	0.00249 (18)	−0.00036 (19)
C12	0.0143 (3)	0.0185 (3)	0.0249 (4)	−0.0031 (2)	0.0062 (3)	−0.0015 (3)

### Geometric parameters (Å, º)

**Table:**

S1—C1	1.7279 (8)	C6—C1	1.4152 (10)
S1—C7	1.7480 (7)	C8—C9	1.4379 (11)
S2—C12	1.7111 (10)	C8—H8	0.9500
S2—C9	1.7213 (8)	C9—C10	1.3830 (11)
N1—C7	1.3058 (10)	C10—C11	1.4183 (13)
N1—C6	1.3831 (10)	C10—H10	0.9500
N2—C8	1.2904 (10)	C11—C12	1.3694 (15)
N2—C7	1.3879 (10)	C11—H11	0.9500
C4—C5	1.3844 (12)	C3—C2	1.3880 (12)
C4—C3	1.4091 (13)	C3—H3	0.9500
C4—H4	0.9500	C2—C1	1.3966 (11)
C5—C6	1.4055 (11)	C2—H2	0.9500
C5—H5	0.9500	C12—H12	0.9500
			
C1—S1—C7	88.76 (4)	C10—C9—S2	111.59 (6)
C12—S2—C9	91.61 (4)	C8—C9—S2	120.57 (6)
C7—N1—C6	109.46 (6)	C9—C10—C11	112.06 (8)
C8—N2—C7	118.12 (7)	C9—C10—H10	124.0
C5—C4—C3	121.06 (8)	C11—C10—H10	124.0
C5—C4—H4	119.5	C12—C11—C10	112.47 (8)
C3—C4—H4	119.5	C12—C11—H11	123.8
C4—C5—C6	118.89 (7)	C10—C11—H11	123.8
C4—C5—H5	120.6	C2—C3—C4	121.13 (8)
C6—C5—H5	120.6	C2—C3—H3	119.4
N1—C6—C5	125.08 (7)	C4—C3—H3	119.4
N1—C6—C1	115.64 (7)	C3—C2—C1	117.74 (8)
C5—C6—C1	119.28 (7)	C3—C2—H2	121.1
N1—C7—N2	127.91 (7)	C1—C2—H2	121.1
N1—C7—S1	116.87 (6)	C2—C1—C6	121.90 (7)
N2—C7—S1	115.22 (6)	C2—C1—S1	128.84 (6)
N2—C8—C9	119.77 (7)	C6—C1—S1	109.26 (6)
N2—C8—H8	120.1	C11—C12—S2	112.27 (7)
C9—C8—H8	120.1	C11—C12—H12	123.9
C10—C9—C8	127.83 (7)	S2—C12—H12	123.9
			
C3—C4—C5—C6	0.46 (13)	C8—C9—C10—C11	−179.24 (9)
C7—N1—C6—C5	−179.28 (8)	S2—C9—C10—C11	−0.19 (11)
C7—N1—C6—C1	0.46 (10)	C9—C10—C11—C12	0.20 (13)
C4—C5—C6—N1	178.98 (8)	C5—C4—C3—C2	0.04 (14)
C4—C5—C6—C1	−0.76 (12)	C4—C3—C2—C1	−0.22 (13)
C6—N1—C7—N2	179.97 (8)	C3—C2—C1—C6	−0.10 (12)
C6—N1—C7—S1	−1.01 (9)	C3—C2—C1—S1	−179.40 (7)
C8—N2—C7—N1	−12.23 (13)	N1—C6—C1—C2	−179.17 (7)
C8—N2—C7—S1	168.73 (7)	C5—C6—C1—C2	0.60 (12)
C1—S1—C7—N1	1.00 (7)	N1—C6—C1—S1	0.26 (9)
C1—S1—C7—N2	−179.84 (6)	C5—C6—C1—S1	−179.98 (6)
C7—N2—C8—C9	−179.96 (7)	C7—S1—C1—C2	178.73 (8)
N2—C8—C9—C10	177.50 (9)	C7—S1—C1—C6	−0.64 (6)
N2—C8—C9—S2	−1.47 (12)	C10—C11—C12—S2	−0.12 (12)
C12—S2—C9—C10	0.10 (8)	C9—S2—C12—C11	0.01 (8)
C12—S2—C9—C8	179.23 (7)		

### Hydrogen-bond geometry (Å, º)

**Table:**

*D*—H···*A*	*D*—H	H···*A*	*D*···*A*	*D*—H···*A*
C11—H11···S2^i^	0.95	2.92	3.517 (1)	122 (1)

Symmetry code: (i) *x*, −*y*−1, *z*−1/2.
